# Physician-related barriers to communication and patient- and family-centred decision-making towards the end of life in intensive care: a systematic review

**DOI:** 10.1186/s13054-014-0604-z

**Published:** 2014-11-18

**Authors:** Mieke Visser, Luc Deliens, Dirk Houttekier

**Affiliations:** End-of-Life Care Research Group, Vrije Universiteit Brussel (VUB) and Ghent University, Laarbeeklaan 103, B-1090 Brussels, Belgium; Department of Medical Oncology, Ghent University, De Pintelaan 185, B-9000 Ghent, Belgium

## Abstract

**Introduction:**

Although many terminally ill people are admitted to an intensive care unit (ICU) at the end of life, their care is often inadequate because of poor communication by physicians and lack of patient- and family-centred care. The aim of this systematic literature review was to describe physician-related barriers to adequate communication within the team and with patients and families, as well as barriers to patient- and family-centred decision-making, towards the end of life in the ICU. We base our discussion and evaluation on the quality indicators for end-of-life care in the ICU developed by the Robert Wood Johnson Foundation Critical Care End-of-Life Peer Workgroup.

**Method:**

Four electronic databases (MEDLINE, Embase, CINAHL and PsycINFO) were searched, using controlled vocabulary and free text words, for potentially relevant records published between 2003 and 2013 in English or Dutch. Studies were included if the authors reported on physician-related and physician-reported barriers to adequate communication and decision-making. Barriers were categorized as being related to physicians’ knowledge, physicians’ attitudes or physicians’ practice. Study quality was assessed using design-specific tools. Evidence for barriers was graded according to the quantity and quality of studies in which the barriers were reported.

**Results:**

Of 2,191 potentially relevant records, 36 studies were withheld for data synthesis. We determined 90 barriers, of which 46 were related to physicians’ attitudes, 24 to physicians’ knowledge and 20 to physicians’ practice. Stronger evidence was found for physicians’ lack of communication training and skills, their attitudes towards death in the ICU, their focus on clinical parameters and their lack of confidence in their own judgment of their patient’s true condition.

**Conclusions:**

We conclude that many physician-related barriers hinder adequate communication and shared decision-making in ICUs. Better physician education and palliative care guidelines are needed to enhance knowledge, attitudes and practice regarding end-of-life care. Patient-, family- and health care system–related barriers need to be examined.

**Electronic supplementary material:**

The online version of this article (doi:10.1186/s13054-014-0604-z) contains supplementary material, which is available to authorized users.

## Introduction

Almost 30% of the Medicare beneficiaries in the United States are admitted to an intensive care unit (ICU) in the last phase of their lives [1]. The conclusion of the SUPPORT study investigators in 1995 was that many patients in ICUs receive unwanted life-sustaining treatments and insufficient palliative care at the end of their lives [2]. In a survey of 82 adult ICUs in 9 European countries plus Israel, shortcomings were perceived in ICU care by 1 in 3 physicians for at least 1 patient on the day of the survey in 2011 [3]. Perceived disproportionate care was the most common shortcoming indicated by physicians, and ‘too much care’ was reported in 89% of those cases. Decisions about end-of-life care were most often reported as being made too late or too infrequently, and nurses as well as physicians were greatly distressed by the perception of inappropriate care.

Death in an ICU is often described as a devastating experience for patients and their families, with patients remaining dependent on intensive life support care, neither dying nor recovering. Expectations are still unrealistically high among patients and their families and also among physicians [4,5]. Continuing life-sustaining treatments without clinical improvement causes suffering to patients and deprives them and their families of palliative care, deprives them and their families of honest prognostic information, and reduces patients’ time to prepare for dying and their families’ time to prepare for bereavement [4,6].

End-of-life care in ICUs is often inadequate because of factors such as lack of communication between patients and health care providers, lack of patient- and family-centred care and lack of emotional and psychosocial support. It is apparent that some of these factors are due to physician-related barriers, many of which have been reported in the scientific literature [6,7]. ICU physicians are unable to provide treatment according to a patient’s wishes when the goals of care and the treatment preferences of the patient are not clear and treatment decisions are not shared with the patient and the patient’s family. As a result, the patient’s quality of life may be harmed. This is why patients and families are currently expressing their wishes for better communication and a larger role in the treatment decision-making process and asking ICU clinicians to respond to their palliative care needs [8].

The Robert Wood Johnson Foundation (RWJF) Critical Care End-of-Life Peer Workgroup identified seven specific domains of ICU palliative care quality, including adequate communication within the team and with patients and families as well as facilitating patient- and family-centred decision-making [9]. In a multicentre study, ICU patients and families expressed strong agreement about the importance of communication and patient- and family-centred decision-making in ICU end-of-life care. Their responses were collected and organised within two domains: communication and decision-making. (1) Adequate communication by clinicians was defined as timely, ongoing, clear, complete, compassionate and focused on the patient’s condition, prognosis and treatment. (2) Adequate patient-focused medical decision-making was defined as being aligned with the patient’s values, care goals, treatment and preferences [6,10]. Within these two domains, 23 quality indicators (QIs) were developed (10 for communication within the team and with patients and their families and 13 for patient- and family-centred decision-making) through a literature review and expert consensus [11].

### Quality indicators for communication

The following are the 10 QIs used to evaluate communication within the team and with patients and their families:

1. Meet as interdisciplinary team to discuss the patient’s condition, clarify goals of treatment, and identify the patient’s and family’s needs and preferences.
2. Address conflicts among the clinical team before meeting with the patient and/or family.
3. Utilize expert clinical, ethical, and spiritual consultants when appropriate.
4. Recognize the adaptations in communication strategy required for patients and families according to the chronic versus acute nature of illness, cultural and spiritual differences, and other influences.
5. Meet with the patient and/or family on a regular basis to review patient’s status and to answer questions.
6. Communicate all information to the patient and family, including distressing news, in a clear, sensitive, unhurried manner, and in an appropriate setting.
7. Clarify the patient’s and family’s understanding of the patients’ condition and goals of care at the beginning and end of each meeting.
8. Designate primary clinical liaison(s) who will communicate with the family daily.
9. Identify a family member who will serve as the contact person for the family.
10. Prepare the patient and family for the dying process.

### Quality indicators for patient- and family-centred decision-making

The following are the 13 QIs used to evaluate patient- and family-centred decision-making:

11. Recognize the patient and family as the unit of care.
12. Assess the patient’s and family’s decision-making style and preferences.
13. Address conflicts in decision making within the family.
14. Assess, together with appropriate clinical consultants, the patient’s capacity to participate in decision making about treatment and document assessment.
15. Initiate advance care planning with the patient and family.
16. Clarify and document the status of the patient’s advance directive.
17. Identify the healthcare proxy or surrogate decision maker.
18. Clarify and document resuscitation orders.
19. Assure patients and families that decision making by the healthcare team will incorporate their preferences.
20. Follow ethical and legal guidelines for patients who lack both capacity and a surrogate decision maker.
21. Establish and document clear, realistic, and appropriate goals of care in consultation with the patient and family.
22. Help the patient and family assess the benefits and burdens of alternative treatment choices as the patient’s condition changes.
23. Forgo life-sustaining treatments in a way that ensures patient and family preferences are elicited and respected.

### Objectives and research questions

Establishing scientific evidence about the barriers that hinder ICU physicians in communication and shared end-of-life decision-making is important to improve the quality of end-of-life care of terminally ill ICU patients. Therefore, the objective of this systematic review was to describe physician-related and physician-reported barriers to the QIs for adequate communication within the team and with patients and families, as well as adequate patient and family-centred decision-making towards the end of life in the ICU as described by the End-of-Life Peer Workgroup of the RWJF.

The following are the specific research questions we sought to answer in this systematic literature review: (1) What are the physician-related and physician-reported barriers to communication within the team and with patients and families in end-of-life care in the ICU according to the 10 QIs for communication within the team and with patients and families in end-of-life care in the ICU, as developed by the RWJF? (2) What are the physician-related and physician-reported barriers to patient- and family-centred decision-making in end-of-life care in the ICU according to the 13 QIs for patient- and family-centred decision-making in end-of-life care in the ICU, as developed by the RWJF?

## Material and methods

### Ethics

Because of the nature of this study, which is a systematic literature review, ethical approval was not required.

### Search strategy

The electronic databases MEDLINE, Embase, CINAHL and PsycINFO were searched for study reports published between 2003 and August 2013 in Dutch or English. We used controlled vocabulary and free text words, including: ‘physicians’ , ‘palliative care’ , ‘advance care planning’ , ‘terminal care’ , ‘terminally ill’ , ‘critical care’ and ‘intensive care units’ (Additional file 1).

### Criteria for eligibility of studies

#### Inclusion criteria

The following were the study inclusion criteria:

- The study addressed a clear research question or objective, and primary collected qualitative or quantitative data were used.
- ICU physicians treating adult patients were reported. Intensive care physicians were defined as attending physicians, critical care fellows, resident physicians or consultants. In studies that included various types of intensive care clinicians, separate results for physicians had to have been reported.
- The report was on physician communication within the team and with patients and families or on patient and family-centred decision-making towards the end of life of patients in an ICU.
- The report was on physician-related barriers to communication and patient and family-centred decision-making as described by the physicians themselves (physician-reported) and not by other caregivers of the ICU-team (for example, nurses), by patients or by proxies or relatives. The focus was on barriers that can be changed; therefore age, sex and background of the physicians were not included as barriers.

#### Exclusion criteria

The following were the study exclusion criteria:

- The report was on medical students, nurses or patients and their families.
- The report was on physicians in nonadult ICUs (for example, neonatal ICU).
- The study had a quality assessment score of 5 or lower assigned independently by MV and DH.

### Study selection

Duplicates of the retrieved records were removed. MV and DH independently examined titles and abstracts of the retrieved records, using a piloted form, to exclude obviously irrelevant records. Disagreement was resolved by consensus, and a third reviewer (LD) was involved for arbitration when necessary.

In the next step of the study selection procedure, the eligibility of retrieved studies was examined independently by MV and DH using a piloted form. When necessary, a third reviewer (LD) was involved for arbitration.

### Data collection

The characteristics of the included studies were extracted to a piloted data extraction form. Physician-related and physician-reported barriers for each of the 10 QIs for communication within the ICU team and with patients and their families, as well as for each of the 13 QIs for patient and family-centred decision-making in end-of-life care in ICU [11], were extracted independently by MV and DH. Barriers were categorized on the basis of whether they related to the physician’s knowledge, attitudes or practice, according to a model developed by Cabana and colleagues [12]. In cases of disagreement, a third reviewer (LD) was involved for arbitration.

### Quality assessment and grading evidence

**Figure 1:**
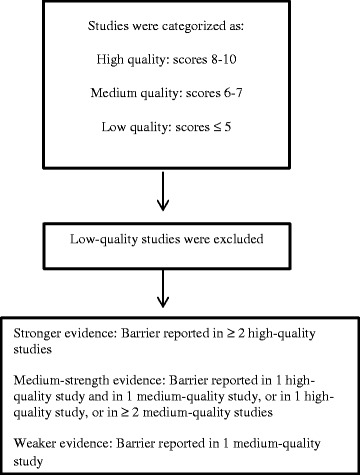
**Quality assessment and grading of evidence.**

The quality of studies with a qualitative research design was assessed using the dedicated tool from the Critical Appraisal Skills Programme [13]. For appraisal of the quantitative studies (all identified eligible quantitative studies were surveys), a survey-specific appraisal tool developed by the Center for Evidence-Based Management was used [14]. Both appraisal tools address the appropriateness of the research method in relation to the study objectives, ethical issues and quality of the data collection and analysis. Quality assessment scores were assigned independently by MV and DH. In cases of disagreement, a third reviewer (LD) was involved for arbitration. For both qualitative and quantitative studies, the total quality assessment scores are presented as scores on a scale from 0 to 10. Studies with assessment scores from 8 to 10 were qualified as high-quality studies; those with scores of 6 to 7 were considered medium-quality studies; and those with scores equal to or lower than 5 were classified as low-quality studies. Low-quality studies were excluded from data synthesis. Barriers reported in two or more high-quality studies qualified as stronger evidence. Barriers reported in one high-quality study and one medium-quality study, or in one high-quality study or in two or more medium-quality studies, were graded as medium evidence. Barriers reported in one medium-quality study were graded as weak evidence (Figure 1).

## Results

### Study selection

**Figure 2:**
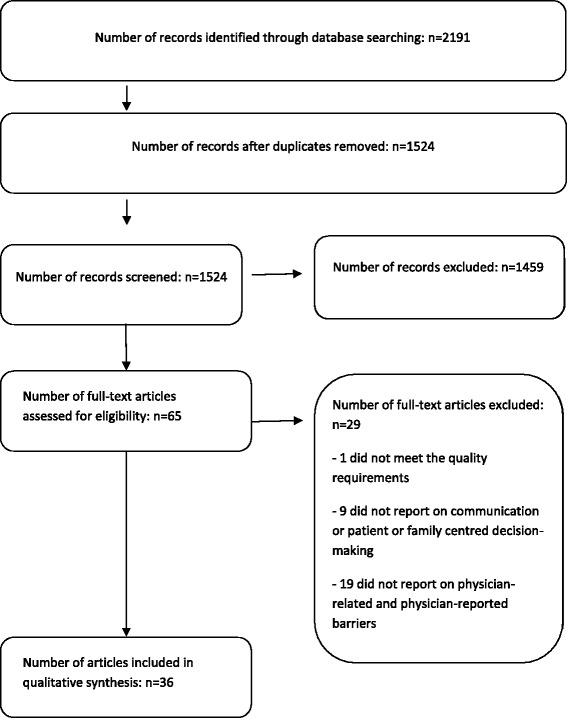
**Study selection process.**

From the electronic database searches, a total of 2,191 records were retrieved. The breakdown was 465 from MEDLINE, 1,285 from Embase, 120 from PsycINFO and 321 from CINAHL (Figure 2). After removal of duplicates (*n* = 667) and obviously irrelevant records (*n* = 1,459), 65 full-text articles were assessed for eligibility. Of those 65, one study did not meet the quality requirements; 9 did not report on physician communication within the team or with patients and families, or on patient- and family-centred decision-making towards the end of life of patients in the ICU; and 19 did not report on physician-related barriers reported by the physicians themselves. Thus, 36 studies met all the inclusion criteria and were used for data synthesis.

### Characteristics and quality assessment of included studies

Of the 36 included studies, 18 were qualitative studies and 18 were surveys (Table 1). Fourteen were conducted in the United States, five were carried out in Canada, 4 included combined results from several European countries, 3 were done in Germany, 2 were conducted in the United Kingdom and 1 each were carried out in Australia, Poland, China, Greece, Austria, Ireland, Hungary and the West Indies. Quality assessment scores varied between 6.5 and 9.5 for qualitative studies and 6 or 7 for quantitative studies.

**Table 1:** **Characteristics and quality assessment of included studies**

**Study (yr) [ref]**	**Country**	**Study objective**	**Study design**	**Participants**	**Quality assessment**
**Qualitative studies**					
Pattison *et al*. (2013) [15]	UK	To explore the meaning of end-of-life care for critically ill cancer patients, their families, oncologists, palliative care specialists, critical care consultants and nurses	Qualitative, phenomenological, in-depth interviews	13 physicians	9
Gutierrez (2012) [16]	USA	To explore the experiences of critical care nurses and physicians with advance directives in an intensive care unit (ICU) to identify the benefits and limitations of advance directives and recommendations for improvement	Descriptive ethnographic study with interviews in a 22-bed medical/surgical ICU in a large community hospital	7 attending physicians, 3 fellow physicians	6.5
Barnato *et al*. (2012) [17]	USA	To explore norms of decision-making regarding life-sustaining treatments at 2 academic medical centres that contribute to opposite extremes of end-of-life ICU use	Mixed-methods study: family meetings, informal and formal interviews, and artefacts	Attending physicians at 2 academic medical centres, patients and family	8
Schenker *et al*. (2012) [18]	USA	To describe whether and how comfort care was presented as an option in family conferences about treatment options, and to assess whether the strength of the physicians’ belief that life support should be withdrawn was associated with the presentation of comfort care	Mixed-methods study of 72 audio-recorded family conferences about end-of-life treatment decisions	Physicians and family	8
Jox *et al*. (2012) [19]	Germany	To explore how clinicians themselves define medical futility, whom they think should assess this, how they justify performing futile treatment and how they communicate futility situations to patients and caregivers	Qualitative mixed-methods approach at a large tertiary referral centre used to analyse protocols of ethics consultations and semistructured interviews	7 physicians	8.5
Baggs *et al*. (2012) [20]	USA	To examine the role of the ‘attending physician’ in four adult ICUs and the consequences of role complexities for clinicians, as well as for patients and their families, particularly in the context of end-of-life decision-making	Ethnographic study in a large academic hospital with surgical, medical, cardiovascular and burn/trauma ICU, including observations of end-of-life discussions and interviews	30 physicians	8.5
Coombs *et al*. (2012) [21]	UK	To identify the challenges for health care professionals when moving from a recovery trajectory to an end-of-life trajectory in intensive care	Semistructured interviews in 2 ICUs in a large university-affiliated hospital in England	13 doctors	9
Ahern *et al*. (2012) [22]	Canada	Interview-based qualitative study conducted to identify what is important to physician trainees in the ICU and infer from this positive educational experiences for physician trainees	Qualitative approach of hermeneutic phenomenology, semistructured interviews	19 critical care physician trainees in their postgraduate years (R4 to R6)	8.5
Gehlbach *et al*. (2011) [23]	USA	Assess the concordance between patients’ code status preferences and their actual code status orders; compare patients/surrogates and their physicians regarding their respective assessments of most important goals of care	Survey, interviews with closed-ended and open-ended questions in a medical ICU of a large academic medical centre	15 physician participants	7.5
Schwarze *et al*. (2010) [24]	USA	To examine the culture and practice of surgeons to assess attitudes and concerns regarding advance directives for their patients who undergo high-risk surgical procedures	Qualitative study in trauma and surgical critical care	10 physicians	7.5
Corke *et al*. (2009) [25]	Australia	To examine attitudes of intensive care doctors to advanced care planning and medical enduring power of attorney	Survey followed by open-ended questions	275 trainees and fellows	7
Sibbald *et al*. (2007) [26]	Canada	To explore how frontline ICU staff defines medically futile care, to discover why they provide it and to identify strategies that might promote a more effective use of ICU resources	Qualitative interviews in 16 ICUs of academic and community hospitals	16 medical directors	8
Beck *et al*. (2008) [27]	Germany	To identify difficulties and uncertainties in making decisions about withholding and withdrawing mechanical ventilation among intensive care physicians	Problem-centred interviews	28 interviewees, 4 consultants, 11 senior registrars, 13 senior house officers (20 of 28 were specialists)	9
Baggs *et al*. (2007) [28]	USA	To clarify unit cultures surrounding end-of-life decision-making in 4 US adult medical and surgical ICUs	Prospective ethnographic study of 4 adult ICUs in which a 6-member research team used participant observations, field notes, and semistructured interviews of health care providers as well as patients and their families	13 physicians	8
White *et al*. (2007) [29]	USA	To determine the nature and extent of shared decision-making about end-of-life treatment in ICUs, which factors are predictive of higher levels of shared decision-making	Mixed-methods study: ICU family conferences in 1 county hospital, 1 university hospital and 2 community hospitals, as well as questionnaires to physicians	35 physicians leading conferences	8.5
Hsieh *et al*. (2006) [30]	USA	To identify inherent tensions that arose during family conferences in the ICU and the communication strategies clinicians used in response	Qualitative content analysis; communication between family members and physicians was analysed using a dialectic perspective in 51 family–clinician conferences in 4 hospitals	36 physicians who led the conferences	8.5
Palda *et al*. (2005) [31]	Canada	To explore the process of the provision of futile care in Canadian ICUs	Survey with closed- and open-ended questions	114 physicians	6.5
West *et al*. (2005) [32]	USA	To identify categories of expressions of nonabandonment in the setting of ICU family conferences concerning withdrawing life-sustaining therapy or the delivery of bad news, and to develop a conceptual model in which nonabandonment is expressed	Qualitative analysis of statements of abandonment during family conferences discussing withholding/withdrawing of treatment	35 physicians leading the conferences	7.5
**Quantitative studies**					
Bülow *et al*. (2012) [33]	European countries (6 countries)	To examine whether religion and religiosity are important to end-of-life decisions and patient autonomy in the ICU	Structured questionnaires in 6 European countries, 143 ICUs	304 physicians	6
Schimmer *et al*. (2012) [34]	Germany	To determine the decision-making process of withholding and/or withdrawing of life-sustaining treatment in cardiac ICUs in Germany	Questionnaire distributed to all heart surgery ICUs (*N* = 79) in Germany	35 clinical directors, 25 senior ICU physicians	6
Kübler *et al*. (2011) [35]	Poland	To analyse the attitudes of ICU physicians regarding decisions to forgo life-sustaining treatment for adult ICU patients	Survey	217 intensive care physicians working in ICUs in Poland	6
Weng *et al*. (2011) [36]	China	To document current attitudes and practices of ICU doctors in China dealing with issues that have strong ethical and moral dimensions; to make comparisons with these attitudes and practices reported by ICU doctors in Hong Kong and Europe	Anonymous, written, structured questionnaire survey	315 participants, representing 54 ICUs in 30 cities in 21 of the 31 regions of China	7
Kranidiotis *et al*. (2010) [37]	Greece	To study the frequency, type and rationale for limiting life support in Greek multidisciplinary ICUs, the clinical and demographic parameters associated with limiting life support, and the participation of relatives in the decision-making process	Prospective observational study, with an anonymous questionnaire in 8 multidisciplinary, general hospital-affiliated ICUs	304 patients and their physicians	7
Schaden *et al*. (2010) [38]	Austria	To explore Austrian intensive care physicians’ experiences with, and their acceptance of, the new advance directives legislation 2 years after enactment	Survey of all ICUs in Austria	139 participants	6
Westphal and McKee (2009) [39]	USA	To examine differences between physicians and nurses regarding knowledge about advance directives and do-not-resuscitate orders, and the personal factors that underlie beliefs and practices related to the use of advance directives and do-not-resuscitate orders	Survey	53 physicians	6
Sprung *et al*. (2008) [40]	European countries (17 countries)	To evaluate physician documentation and the reasoning, considerations and difficulties in end-of-life decision-making in ICUs	Prospective study of end-of-life practices and decisions in consecutive patients who died or were subject to any limitation of life-saving interventions in 37 ICUs in 17 European countries	ICU physicians	6
Collins *et al*. (2006) [41]	Ireland	To study the frequency, rationale and process for withholding and withdrawing life-sustaining treatment in intensive care patients in Ireland	Prospective observational study of all consecutive patients admitted to ICU who died or had life-sustaining treatment limited	Data of 122 patients, documented by physicians	7
Nelson *et al*. (2006) [42]	USA	To improve the understanding of educational needs among residents caring for the critically ill	Survey	184 physicians	7
White *et al*. (2006) [43]	USA	To determine how decisions are made to limit life-sustaining treatment for critically ill patients who lack both decision-making capacity and surrogate decision makers	Prospective longitudinal cohort study	47 physicians of patients without decision-making capacity and without a surrogate	6
Moss *et al*. (2005) [44]	USA	To assess the knowledge, skills and attitudes that physicians and nurses who practice in West Virginia’s ICUs have concerning end-of-life care	Survey	153 physicians	6
Cohen *et al*. (2005) [45]	European Countries (17 countries)	To examine the communication of end-of-life decisions in Europe	Prospective observational study of 4,248 patients who had any limitation of life-sustaining treatment or died in 37 ICUs in 17 countries	Physicians collected data on 4248 patients	7
Élő *et al*. (2005) [46]	Hungary	To study the factors associated with limiting resuscitation in Hungary	Survey	72 doctors	7
Sinuff *et al*. (2004) [47]	Canada, USA, Sweden, Australia	To study the rate of establishing do-not-resuscitate directives, determinants and outcomes of those directives for mechanically ventilated patients	Multicentre observational study	3,099 critically ill patients admitted to 15 ICUs, documentation attending physicians’ clinical judgements	7
Yap *et al*. (2004) [48]	Hong Kong	To examine ethical attitudes of intensive care physicians in Hong Kong	Survey	65 physicians	7
Hariharan *et al*. (2003) [49]	West Indies	To analyse the characteristics of moribund patients in a surgical ICU and highlight the dilemmas inherent in treating such patients	Prospective collection of data from patient records	Data of patients recorded by physicians of surgical ICU	6
Garland and Connors (2007) [50]	Canada	To quantify the influence that ICU staff physicians have on decisions to limit life support for critically ill patients	Data prospectively collected in the 13-bed medical ICU of a 520-bed urban university-affiliated teaching hospital	9 staff physicians	7

### Barriers

All the barriers reported by ICU physicians were categorized by QI as developed by Clarke and colleagues [11] according to barriers related to the knowledge, attitudes and practices of physicians [12]. Ninety different barriers were identified, among which 24 related to physician knowledge (Table 2), 46 to physician attitudes (Table 3) and 20 to physician practice (Table 4). Stronger evidence was found for 8 specific barriers (all related to physician attitudes), medium evidence for 39 and weak evidence for 43.

**Table 2:** **Barriers with regard to physicians’ knowledge**

**Quality indicator no.** ^**a**^	**Barriers with regard to physicians’ knowledge**
General	• Insufficient physician training in communication about end-of-life issues^b^ [42]
	• Clinician reluctance to use opioids or sedatives because of concern about side effects^b^ [42]
	• Lack of education in palliative medicine^b^ [44]
1	• Involvement of surgeons slows down decision-making because they do not understand patient’s situation^c^ [21]
2	• Lack of communication skills of senior medical residents when interacting with colleagues^c^ [22]
5	• No familiarity with skilled and timely communication^c^ [26]
10	• Not taught how to recognize that a person is about to die, no awareness of the process of dying^c^ [21]
	• Unrealistic expectations by clinicians about patient prognosis or effectiveness of ICU treatment^b^ [42]
16	• Physician uncertainty about the legal details of advance directives^b^ [38]
	• Physician lack of physician experience with advance directives^b^ [38]
21	• Lack of familiarity to make a prognosis^c^ [15]
	• Not knowing how to deal with ‘feeling helpless’ with families pressuring ICU teams to withhold treatment or when family members are upset about aggressiveness of care provided to their unwilling loved one^c^ [22]
	• Uncertainty concerning the services provided by local hospice programs and whom to refer to hospices^b^ [44]
	• No awareness of professional or local guidelines that related to provision of futile care^b^ [31]
	• Insufficient training in communication with patients and their families^b^ [31]
	• Lack of discussion of ethical issues in medical programmes; lack of knowledge of ethical issues concerning end-of-life decisions^b^ [48]
22	• No familiarity with defining futility and how to communicate futility to patients and their families^c^ [19]
	• No knowledge of management of critical illness by referring specialists; confounding factors in decision-making^c^ [21]
23	• Conditioned that doing nothing or withdrawing treatment is not helping patient^c^ [19]
	• No familiarity with legal framework regarding end-of-life decisions, wrong conception that law prohibits withdrawal of mechanical ventilation^c^ [27,36]
	• No awareness of end-of-life care guidelines^c^ [27]
	• Not being at ease in talking to patients and their families about limitations of therapy^b^ [36]
	• No familiarity with end-of-life decision-making (‘good prognosis’ and ‘give it a go’ often said because of no familiarity with end-of-life decision-making)^b^ [25]
	• Insufficient clinician training in techniques for forgoing life-sustaining treatment without causing patient suffering^b^ [42]

^a^Quality indicators for adequate communication and decision-making in the ICU as developed by Clarke and colleagues [11] and as outlined above in the Introduction. ^b^Barriers for which weak evidence was found. ^c^Barriers for which medium-quality evidence was found.

**Table 3:** **Barriers with regard to physicians’ attitudes**

**Quality indicator no.** ^**a**^	**Barriers with regard to physicians’ attitudes**
1	• Lack of consensus among the treating team in making end-of-life decisions, surgeons in the ICU do not want to give responsibility to other members of the clinical team, looking only at the small percentage of patients who survive, and one physician could push for futile treatment looking only at a small aspect of the patient’s overall condition^b^ [20,26]
	• Perception by the critical care attending physician that the consulting specialist controls life-sustaining treatment decision-making^c^ [17]
	• Physicians are overly sure of making the right decision themselves; they do not include patients in care decisions and consensus development^c^ [21,37]
2	• Conflicting opinions of different attending physicians about prognosis and treatment and about recognition that death is a potential reality ^b^ [15,20]
	Surgeon’s disagreement with other consultants to accept futility treatment^d^ [49]
5	• Negative attitude towards relatives who want limitation of treatment^d^ [37]
6	• Family is thought not to understand end-of-life practice, family was considered not available, or physicians found discussion with relatives unnecessary^d^ [37]
10	• Palliative care input was limited to the very end of life, ‘death is not usually expected’, and narrow interpretation of when a patient is dying (that is, that a patient whose vital signs cannot be maintained despite maximal life-sustaining treatment is dying^b^ [15,17]
	• Physicians sometimes use language that seems to imply abandonment of their patients during the end-of-life decision-making process, as if withdrawal is the sole responsibility of the family, without mentioning another mode of care^d^ [32]
11	• Uneasiness in dealing with surrogate decision maker^c^ [22]
	• Family is thought not to understand, family was not available, or physician found discussion unnecessary^c^ [37,45]
15	• Negative opinion of advance directives, often perceived as not preventing unwanted aggressive treatment (because of lack of communication with relatives) and lacking a level of specificity necessary to facilitate decision-making^d^ [16]
	• Physicians’ own ethical values regarding advance directives^d^ [38]
18	• Physicians consider do-not-resuscitate orders paperwork, slow, and not applicable to situations related to dying at the ICU^c^ [28]
	• Physicians are not aware of patients’ preferences regarding do-not-resuscitate orders^d^ [23]
	• Physicians believe that do-not-resuscitate orders should not be applied^d^ [36]
	• Most physicians only discuss do-not-resuscitate order when the prognosis is poor or when the patient’s condition deteriorates^c^ [39,47]
	• Family dynamics and legal concerns were the most important concerns affecting physicians’ decision to write or obtain a do-not-resuscitate order^d^ [39]
	• The most important factor influencing do-not-resuscitate decisions was the opinion of the head of the department or the doctor in charge of the patient’s care, not the wishes of the patient and/or the patient’s family^d^ [46]
21	• No acceptance that the patient is dying; opinion that life should be the foremost concern in end-of-life decision making and that patient’s goal of care is to survive (surgeons); physicians cannot let patients die: “They regard life at any cost to be a success” (comment physician)^b^ [21,28,31,35]
	• Conflicting opinion about prognosis, medical uncertainty and focus on narrow physiologic objectives without recognition that the condition of the patient becomes terminal, reaching a point of futility with prolongation of dying; these are barriers limiting the amount of time left for appropriate decision-making^b^ [15-17]
	• Surgeons in the team want to continue life-sustaining treatment; they do not accept that they cannot go any further; they do not consider end-of-life discussions in the surgical ICU, which take place later in the patient’s illness trajectory, often in a critical atmosphere^b^ [19,20]
	• Physicians are sure of making the right decisions themselves and do not include patients in care decisions and consensus development^c^ [21,37]
	• Think that families do not understand end-of-life practices, that families are not available, or that discussions about goals of care are unnecessary^c^ [37,45]
	• Think that time spent with family wastes time and energy when families want continuation of aggressive treatment or when there is disagreement or extended hesitation over a decision^c^ [22]
	• No appropriate communication strategy, no information-seeking, but instead arguing with patient and/or the patient’s family or avoiding discussions with them as decision-centred strategy^c^ [30]
	• Not eliciting of family’s wishes or assessment of family’s understanding of information; the family is often more told than asked about the nature and context of end-of-life decisions^c^ [29,45]
	• Feeling of loss of control of referred patients and not believing in giving up on patients are reasons not to refer patients to hospice^d^ [44]
	• No recognition of patients’ goals of care^d^ [23]
22	• Physicians find it easier to carry on with treatment than to discuss alternative goals of care^c^ [21]
	• Surgeons consider informed consent documentation as a contract for potentially burdensome postoperative therapy after a difficult operation (for example, transplant, neurosurgery)^d^ [24]
23	• Concerns about omission of life-sustaining treatment are larger (missing something treatable, fear of doing something wrong or limiting life-sustaining treatment for a patient who might survive) than concerns about harm of administering life-sustaining treatment (such as iatrogenic harms, prolonging dying, and treating patients against their preferences)^b^ [17,19]
	• Having end-of-life care discussions or engaging in shared decision-making with the patient and/or the patient’s family is considered only when the physician believes that life support should be withdrawn^b^ [18,29]
	• Physicians’ concerns about potential legal action taken by families due to forgoing life-sustaining treatment; therefore, they follow families’ wishes, even after reading patients’ advance directives and even when the medical staff uniformly feels that it is not medically appropriate because treatment is futile^c^ [16,26,36,39,42]
	• Physicians prefer their own ideas about the best interests of the patient, are more focused on medical technical parameters concerning withholding or withdrawing therapy, and continue treatment, not respecting the patient’s and/or the patient’s family’s wishes or the patient’s living will to stop treatment^c^ [27,33,34,40]
	• Diagnostic uncertainty or potential for reversibility of illness is justification for continuation of treatment against the instructions in the patient’s medical enduring power of attorney or the patient’s wishes for palliation^c^ [25,37]
	• Unresponsiveness to treatment already offered is the main factor influencing the physician’s decision to withhold or withdraw therapy, not the patient’s and/or the patient’s family’s request^c^ [37,40,41]
	• Doubts about the validity of the patient’s wishes expressed earlier^c^ [25,27]
	• Less respect for patients’ wishes by surgeons compared to other ICU physicians^c^ [28]
	• Feeling of betrayal, unhappiness, disappointment and even culpability when family member confronts physician with advance directives in the setting of prolonged life-sustaining treatment^d^ [24]
	• The treating physician considers death in the ICU as a personal failure^d^ [24]
	• Physician’s distrust of the health care proxy’s motivation to request forgoing life-sustaining treatment and the family’s underlying preferences^d^ [25]
	• Physician's distrust concerning the timing of the completion of the advance directive^d^ [25]
	• Physician’s conception that medical enduring power of attorney and advance directives provide indications or guidelines rather than a decision that has to be respected^d^ [25]
	• Legal concerns or disagreements with other physicians about whether it is appropriate to write a do-not-resuscitate order or withdraw treatment from patients who lack decision-making capacity and do not have a surrogate decision maker^d^ [43]
	• Personal values and beliefs of intensivists, more than comorbidities or the type of acute illness, are barriers to forgoing life-sustaining treatment^d^ [50]

^a^Quality indicators for adequate communication and decision-making in the ICU as developed by Clarke and colleagues [11] and as outlined above in the Introduction. ^b^Barriers for which strong evidence was found. ^c^Barriers for which medium-quality evidence was found. ^d^Barriers for which weak evidence was found.

**Table 4:** **Barriers with respect to physicians’ practice**

**Quality indicator no.** ^**a**^	**Barriers with respect to physicians’ practice**
General	• Competing demands for clinicians’ time^b^ [42]
1	• Unavailability of attending physicians due to rotation systems^c^ [22]
2	• Hierarchy under physicians is a barrier to their solving problems within the team before talking to the patient^c^ [22]
	• Individual physicians’ lack of holistic views^c^ [26]
7	• Physicians do not routinely check that family members understand the information they are given and do not discuss the family’s role in decision-making^c^ [29]
10	• Low confidence in taking responsibility; physicians do not refer patients to hospice care, because the patient or the patient’s family does not accept that the patient is dying^b^ [44]
15	• Not actively recommending the creation of an advance directive^b^ [38]
21	• Low confidence in taking responsibility; the physician does not take responsibility for collaborative decision-making with the dying patient and thus leaves the patient to die as if the patient has decided when to die^c^ [15]
	• Low confidence in taking responsibility; the physician considers family requests for continued futile treatment as a mandate and not as part of a normal communication and decision-making process^c^ [17,31]
	• Low confidence in taking responsibility; the physician externalizes control of decision-making to patients, their families and specialists, who they believe expect aggressive treatment^c^ [17]
	• Postponing decision-making until all treatment options are exhausted, until the last moment (surgeons)^c^ [28]
	• No use of professional or local guidelines related to the provision of futile care^b^ [31]
23	• Lack of time and information are reasons to initiate life support, resulting in futile treatment^c^ [26]
	• Continuation of aggressive treatment is justified, because of lot of money is already invested in the patient, and availability of resources^c^ [17]
	• Aggressive care deemed to be appropriate because of no awareness among providers of existence of advance directive or living will^b^ [16]
	• Low confidence in taking responsibility; the rate of withholding and withdrawing therapy was reduced based upon family’s wishes^b^ [35]
	• Considering withholding and withdrawing decisions inappropriately delayed^b^ [37]
	• No support of an internal multidisciplinary committee or professional policies in cases involving patients who do not have decision-making capacity or a surrogate^b^ [43]
	• Low confidence in taking responsibility; when the patient’s family insists that everything should be done for a patient with a poor prognosis, physicians are less inclined to withdraw treatment than when the family insists on limitation of therapy^b^ [48]
	• Low confidence in taking responsibility; high hopes of the family and their consistent requests to the surgeons contribute to the continuation of therapy which was considered futile by at least two consultants^b^ [49]

^a^Quality indicators for adequate communication and decision-making in ICU as developed by Clarke and colleagues [11] and as outlined above in the Introduction. ^b^Barriers for which weak evidence was found. ^c^Barriers for which medium-quality evidence was found.

#### Barriers with regard to physicians’ knowledge

Barriers with regard to physicians’ knowledge were identified for 8 of the 23 QIs. Because not many barriers were identified per QI, almost identical barriers with low- or medium-quality evidence were compared and combined across the QIs.

Across QIs, strong evidence was found for the barrier of lack of communication training and skills in end-of-life discussions in general [42] and for QIs 2, 5, 21, 22 and 23 [19,22,26,31,36], including how to communicate to patients and their families the futility of further treatment.

*Meet as interdisciplinary team to discuss the patient’s condition, clarify goals of treatment, and identify the patient’s and family’s needs and preferences (QI 1)*

Medium-quality evidence was found for the barrier that, owing to the hierarchy of the system, surgeons who are not fully aware of the patient’s actual condition (that is, that further treatment may be futile) can slow down the team’s decision-making process [21].

*Preparing the patient and family for the dying process (QI 10)*

One barrier found was that physicians are not taught how to recognise that a person is about to die (medium-quality evidence) [21] and have unrealistically high expectations about the patient’s prognosis and effectiveness of ICU treatment (weak evidence) [42].

*Clarify and document the status of the patient’s advance directive (QI 16)*

Physicians are uncertain about the legal standing of, and have no experience with, advance directives (weak evidence) [38].

*Establish and document clear, realistic, and appropriate goals of care in consultation with the patient and family (QI 21)*

Lack of familiarity with how to make a prognosis (medium-quality evidence) [15] and not knowing how to relate to families who pressure them to continue treatment or are upset by the aggressiveness of treatment given against their wishes were identified as barriers (medium-quality evidence) [22].

*Help the patient and family assess the benefits and burdens of alternative treatment choices as the patient’s condition changes (QI 22)*

Referring specialists are not familiar with the management of the critical illnesses of ICU patients, which can lead to difficulties when changes in management have to be made (medium-quality evidence) [21].

*Forgo life-sustaining treatments in a way that ensures patient and family preferences are elicited and respected (QI 23)*

Not being aware of the laws applying to do-not-resuscitate status and the limitation of life-sustaining treatment or withdrawal of treatment (medium-quality evidence) [27,36], not being aware of end-of-life guidelines (medium-quality evidence) [27] and being conditioned to treat for recovery rather than to do nothing (medium-quality evidence) [19] were all identified as barriers.

#### Barriers with regard to physicians’ attitudes

Barriers with regard to physicians’ attitudes were identified for 11 of the 23 QIs. Strong evidence was found for eight specific barriers.

*Meet as interdisciplinary team to discuss the patient’s condition, clarify goals of treatment, and identify the patient’s and family’s needs and preferences (QI 1)*

There is a danger of lack of consensus among the treating team when the focus of the surgeon is on the small percentage of patients who will survive because of their treatment and not on the greater percentage who will not, as well as when ICU physicians focus on the particular aspect of the patient’s condition which comes under their remit rather than on their overall condition and thus do not want to pass responsibility to other members of the clinical team who may have a more holistic perception of the patient’s condition (strong evidence) [20,26]. Medium-strength evidence was found for the barriers that physicians are overly sure of making the right decision themselves and that they do not include nurses’ opinions and patients’ needs in making care decisions and developing a consensus [21,37].

*Address conflicts among the clinical team before meeting with the patient and/or family (QI 2)*

Disagreement and conflicting opinions about the patient’s prognosis and treatment and the imminence of death by different attending physicians was found to be a barrier (strong evidence) [15,20].

*Prepare the patient and family for the dying process (QI 10)*

A narrow interpretation of when a patient is dying, that is, when vital signs cannot be maintained despite maximal life-sustaining treatment and the consideration of palliative care as being appropriate only for the very end of life were both identified as barriers (strong evidence) [15,17].

*Recognize the patient and family as the unit of care (QI 11)*

Medium-strength evidence was found for the barriers in which physicians feel uneasy dealing with surrogate decision makers [22] because they think that the family will not understand and feel that end-of-life discussions with relatives unnecessary [37,45].

*Initiate advance care planning with the patient and family (QI 15)*

Identified as barriers were (1) the physicians’ personal ethical values and (2) their negative opinions of advance directives, which they consider inapplicable in emergencies and lacking a level of specificity (weak evidence) [16,38].

*Clarify and document resuscitation orders (QI 18)*

We found medium-level evidence for the barrier that physicians discuss do-not-resuscitate orders only when the patient’s prognosis is poor [39,47], and the most important factor influencing the decision to write a do-not-resuscitate order is the physician’s opinion and not the wishes of the patient or the patient’s family to stop treatment (weak evidence) [46]. The family’s wish that a do-not-resuscitate order not be written and their concerns about the legality of the order are important in influencing a physician’s decision (weak evidence) [39]. A physician’s beliefs that do-not-resuscitate orders should not be applied (weak evidence) [36] and that do-not-resuscitate orders are a lot of paperwork or are not applicable to the situation of the dying patient (medium-quality evidence) [28] were also considered to be barriers.

*Establish and document clear, realistic, and appropriate goals of care in consultation with the patient and family (QI 21)*

We found strong evidence that physicians’ personal beliefs and values can hinder the process of establishing and documenting clear, realistic and appropriate goals of care with the patient and family. Surgeons are trained to believe that the goal of treatment is the patient’s survival. Physicians tend not to accept that a patient is dying and believe that the patient’s life should be saved [21,28,31,35]. We also found strong evidence for the barrier that surgeons in particular want to continue life-sustaining treatment and that end-of-life discussions take place later in the surgical ICU than in the medical ICU [19,20]. Strong evidence was found that physicians’ conflicting opinions about the patient’s prognosis and their focus on narrow physiological objectives, without recognition that the condition of the patient has become terminal, are barriers to timely end-of-life discussions [15-17].

Physicians are sure of making the right decisions themselves without including patients in care decisions and without consensus development (medium-level evidence) [21,37] and believe that families do not understand end-of-life practices, such that discussions about the goals of care are not necessary (medium-level evidence) [37,45]. Time spent with the family is considered as wasted when the family insists on futile treatment (medium-level evidence) [22]. Evidence was also found for the barrier that physicians do not use appropriate communication strategies in discussions with the patient or the patient’s family, but either argue or avoid discussions (medium-level evidence) [30] or inform the patient or the patient’s family only about the nature and context of the end-of-life decision and do not ask them about their wishes and preferences [29,45].

Further, physicians do not recognize patients’ goals of care, which are more related to quality of life related than to physicians’ goals of their living longer (weak evidence) [23]. The physician’s feeling of loss of control of referred patients and the physician’s perceptions that doing nothing equals giving up on a patient are seen as reasons not to refer patients to hospices (weak evidence) [44].

*Help the patient and family assess the benefits and burdens of alternative treatment choices as the patient’s condition changes (QI 22)*

Physicians find it easier to carry on with treatment than to discuss alternative goals of care (medium-strength evidence) [21], and surgeons consider informed consent to be a contract for potentially burdensome postoperative therapy after a difficult operation (for example, organ transplant, neurosurgery) (weak evidence) [24].

*Forgo life-sustaining treatments in a way that ensures patient and family preferences are elicited and respected (QI 23)*

Strong evidence was found for the barrier that physicians are more concerned that, by abandoning life-sustaining treatment, they might miss something which is treatable than that they might harm patients by prolonging life-sustaining treatment and the dying process or by treating patients against patients’ preferences [17,19]. Strong evidence was also found for the barrier that only if physicians themselves believe that life support should be withdrawn will they consider end-of-life discussions and shared decision-making with the patient and/or the patient’s family [18,29].

Medium-strength evidence was found that physicians prefer their own ideas of what is in the best interest of the patient, focusing instead on clinical and technical parameters to decide on withholding or withdrawing therapy, and do not respect the wishes of the patient or the patient’s family to stop therapy, even when there is a living will [27,33,34,40]. Further, medium-strength evidence was found for the barrier that, even when the team confirms that treatment is futile and inappropriate, physicians follow the family’s wishes when the family wants to continue futile treatment out of concerns about legal action [16,26,36,39,42]. Medium-strength evidence was also found for the barrier that uncertainty regarding the patient’s prognosis and the potential for reversibility of the patient’s illness are used as justification for continuation of treatment against the patient’s or the legal proxy’s wishes for palliation [25,37]. We also found medium-strength evidence for the barriers that the main factor that influences the physician to forgo therapy is the patient’s unresponsiveness to treatment already offered and not the requests of the patient or the patient’s family [37,40,41]. Barriers to respecting an advance directives and medical enduring power of attorney expressing the patient’s wish to forgo treatment are that physicians have doubts about the validity of advance directives (medium-quality evidence) [25,27], distrust concerning the timeliness of an advance directive, feelings of betrayal when confronted with an advance directive (weak evidence) [24,25] and a perception that the medical enduring power of attorney and advance directive provide indications or guidelines rather than a decision that has to be respected (weak evidence) [25].

#### Barriers with regard to physicians’ practice

Barriers with regard to physicians’ practice were identified for 7 of the 23 QIs. Lack of confidence in taking responsibility for communication and patient- and family-centred decision-making was a barrier identified for QIs 10, 21 and 23.

*Meet as interdisciplinary team to discuss the patient’s condition, clarify goals of treatment, and identify the patient’s and family’s needs and preferences (QI 1)*

Unavailability of attending physicians due to the rotation system was found to be a barrier with medium-strength evidence [22].

*Address conflicts among the clinical team before meeting with the patient and/or family (QI 2)*

Hierarchy within the team was seen as a barrier to solving problems before talking to the patient (medium-level evidence) [22]. Individual physicians’ lack of a holistic view was also seen as a barrier (medium-quality evidence) [26].

*Clarify the patient’s and family’s understanding of the patient’s condition and goals of care at the beginning and end of each meeting (QI 7)*

Physicians do not routinely check that family members understand the information they are given and fail to discuss the family’s role in decision-making (medium-level evidence) [29].

*Prepare the patient and family for the dying process (QI 10)*

Lack of confidence in taking responsibility for referring a patient to a hospice, the physician does not do so because the family does not accept that the patient is dying (weak evidence) [44].

*Establish and document clear, realistic, and appropriate goals of care in consultation with the patient and family (QI 21)*

We found medium-strength evidence for physicians’ lack of confidence in taking responsibility as a barrier to decision-making with the dying patient; instead, physicians continue life-sustaining treatments until the patient dies [15]. We found medium-strength evidence that ICU surgeons postpone decision-making until all treatment options have been exhausted, until the very last moment [28]. Medium-strength evidence was also found for other barriers related to lack of confidence to take responsibility. Physicians consider a family’s request for futile treatment as a mandate and not as part of normal communication in the decision-making process [17,31], and they externalize control for decision-making to patients, their families and consulting specialists, who they believe expect aggressive treatment [17].

*Forgo life-sustaining treatments in a way that ensures patient and family preferences are elicited and respected (QI 23)*

Medium-strength evidence was found for the barrier that lack of time and information are reasons to continue therapy, as is money already invested in the patient’s care and the availability of resources (medium-strength evidence) [17].

Weak evidence was found for lack of confidence in taking responsibility; the rate of withholding or withdrawing therapy was reduced at the family’s request [35]. Physicians are less inclined to withdraw treatment when the family insists that everything should be done than when the family asks for limited therapy [48]. A family’s high expectations and consequent requests to the surgeon contribute to the continuation of therapy considered futile by at least two other consultants (weak evidence) [49].

## Discussion

The RWJF Critical Care End-of-Life Peer Workgroup has identified seven specific domains of ICU palliative care quality comprising adequate communication within the team and with patients and families and patient- and family-centred decision-making. However, no systematic description and analysis of barriers to adequate communication and decision-making have been undertaken before. To our knowledge, our present review is the first time that self-reported barriers to providing quality end-of-life care in these two domains have been identified for the main professional actor in the ICU—the ICU physician.

In 36 empirical studies, we identified 90 different physician-related barriers to adequate communication and patient- and family-centred decision-making towards the end of the patient’s life in intensive care as they relate to ICU physicians’ knowledge and skills, attitudes and practices. With respect to physicians’ knowledge and skills, strong evidence was found for physicians’ lack of communication training and skills in general, including communication with colleagues, and in particular regarding the communication of the futility of further treatment to the patient and the patient’s family. Among barriers with strong evidence related to the attitudes of physicians, we found the lack of consensus among the treating team in end-of-life decision-making, when surgeons and physicians focus only on the small percentage of patients who will survive and do not want to share responsibility with other members of the clinical team, to be a barrier to interdisciplinary team discussions. Disagreement between team members and conflicting opinions about the patient’s prognosis and treatment and about the futility of treatment are all barriers to addressing conflict within the team. The narrow interpretation by physicians of when a patient is actually dying, preventing the provision of palliative care until the last moment, is a barrier to preparing the patient and the patient’s family for the dying process. The personal beliefs and values of physicians hinder the process of establishing and documenting clear, realistic and appropriate goals of care with the patient and the patient’s family. Surgeons and physicians are trained to believe the goal of treatment is to save the patient’s life and therefore to resist acknowledging that the patient is dying. Regarding the decision to forgo life-sustaining therapy, we found that physicians were more worried that they might miss something treatable than that they might harm patients with the prolongation of treatment and the dying process, even when this was against the patient’s preferences. They tended to favour their own views of what is in the best interest of the patient, focusing on clinical and technical parameters rather than respecting the wishes of the patient and the patient’s family to forgo treatment. Related to physicians’ practice, we found that physicians often report that they lack the confidence to take responsibility for the dying patient and therefore postpone decision-making about withholding or withdrawing of treatment until all treatment options have been exhausted, thus continuing treatment until the patient dies.

The results of our review indicate that the lack of communication skills among physicians, the weakness of their skills in prognostic estimation and their lack of knowledge about the relevant legal framework are all barriers to the provision of good end-of-life care to patients in the ICU. The barriers we found with regard to physicians’ attitudes demonstrate that physicians often see their job as more to save patients’ lives than to let patients die in the best possible way. When physicians have to make decisions on the withholding or withdrawing of life-sustaining treatment, they favour their own ideas and focus on narrow physiological, technical and clinical parameters rather than on asking patients and patients’ families about preferences regarding treatment. This suggests a lack of a holistic view of the patient’s situation and prevents an understanding of what the patient sees as being in his or her own best interests. Because physicians are inclined to continue providing life-sustaining treatment, they ignore the harm that this may inflict upon the patient, to ignore the wishes of the patient and the patient’s family to stop treatment, and to ignore the fact that the patient is actually dying. This means that timely end-of-life discussions are not possible and that the patient’s wishes and preferences for the last phase of life are not respected, and thus their suffering continues. Palliative care, if it is provided at all, is suspended until the very last moment.

In team meetings and conflicts, when different team members have different opinions about continuation of life-sustaining treatment, the opinion of the consultant that treatment should be continued takes precedence, even when other team members consider such treatment to be futile. This authoritarian attitude is a barrier that prevents the provision of good end-of-life care to patients in the ICU. When the patient’s family wants futile treatment to be continued, physicians have concerns about fulfilling their legal obligations and follow the family’s wishes. However, when the patient and the patient’s family want to stop therapy, physicians often continue it even in many countries in which the law recognizes the right of the patient to refuse treatment. Enforcement of these laws seems to be deficient and should be strengthened. Physician-related barriers to practice reveal that physicians lack confidence in their own judgment that treatment is futile and postpone decision-making about withdrawal of life-sustaining treatment until all treatment options have been exhausted and consider the family’s request to continue futile life-sustaining therapy as mandatory. They do not consider communication and decision-making in the last phase of the patient’s life as a normal process whereby the wishes of the patient’s family are discussed earlier and thus during the last moments of the patient’s life consensus can be achieved about the futility of the life-sustaining treatment.

We compared our findings with the findings of studies on barriers to communication in end-of-life care or advance care planning perceived by general practitioners, to find out if those barriers reported by ICU physicians are specific to practice in ICUs. Some barriers were in line with the findings of a systematic review by Slort and colleagues [51] on barriers for general practitioners, such as ‘general physicians’ (GPs’) lack of availability, and knowledge about palliative care, unpredictability of the patient’s clinical course, not talking honestly to patients about end-of-life care issues and practice barriers, for example, difficulty in dealing with patients in denial and not spontaneously taking the initiative to contact patients. In a systematic review by De Vleminck and colleagues on GP barriers to engaging in advance care planning, doubt regarding the content and practical availability of living wills was identified as a barrier to initiating advance care planning [52]. However, the backgrounds of GPs and ICU physicians are quite different. The GPs are better trained in communication with the patient and in taking a holistic approach and might have a long-standing relationship with the patient and the patient’s family. ICU physicians do not have that long-standing relationship but are confronted much more often than GPs with patients in the last phase of life, and these patients are often in a critical condition in which communication is already difficult. The findings of these two reviews [51,52] and our present review emphasize all the more the need to address the ICU physician–related barriers to communication with patients towards the end of life.

Our review has some limitations. It done during the past ten years, and we excluded studies published before 2003. During the past ten years, however, much attention in the public and professional domains has been given to end-of-life care, especially in the ICU, and we expect that the most important barriers are included in the studies published during this period. The study was limited to studies published in the English or Dutch language, so there may be studies from other countries that we did not include. Moreover, by limiting this review to barriers related to and reported by physicians, we excluded barriers perceived by nurses, patients, patients’ family members and other care providers, as well as structural and institutional factors, so an overall perspective of barriers in the two quality domains could not be achieved.

Our results suggest that ICU physicians need to be trained in using a holistic approach to treating patients at the end of life and in communication competencies. Undergraduate and postgraduate medical educators already see training in communication skills as essential. They also view training in the legal framework and ethical principles of health care as important, as well as defining the role and competencies of the physician who cares for patients towards the end of life; however, such training often is not fully implemented. Palliative care guidelines and support teams in ICUs could help the ICU team to trigger a learning process in caring for patients towards the end of life without the intention of handing over such care completely to the palliative care team. Such a palliative care support team could also help the ICU team, by meeting them together as an interdisciplinary team to address conflicts of opinion.

Further research is needed to investigate interventions and to develop guidelines and protocols helpful to overcoming ICU physician–related barriers regarding adequate communication and patient-centred decision-making towards the end of life. Also, research is needed regarding barriers related to and reported by patients, their family members, and other care providers, as well as with regard to structural and institutional barriers.

## Conclusions

We identified 90 different barriers reported by ICU physicians themselves that stand in their way to providing quality end-of-life care with respect to communication and decision-making. These barriers are related to physicians’ knowledge, attitudes and practices. It is necessary to address these different barriers to improve the quality of end-of-life care for patients and their families in the ICU. In addition to the perspectives of the physicians, it is important to examine the barriers related to and reported by patients and patients’ relatives, as well as other health care providers, in the ICU.

## Key messages

- Ninety different physician-related barriers for quality communication and patient- and family-centred decision-making in end-of-life care in ICUs were identified. These barriers were related to physicians’ knowledge, attitudes and practices.
- Regarding physicians’ knowledge, strong evidence was found for physicians’ lack of training in skills related to communication with patients, patients’ families and physicians’ colleagues, including communication of the futility of further treatment, as a barrier.
- Regarding physicians’ attitudes, strong evidence was found for multiple barriers. These barriers are physicians’ focus on the small percentage of patients who will survive and not on the larger number who will not and therefore will have to undergo intensive care treatment before they die; physicians’ personal beliefs and values and their focus on only clinical and technical parameters; physicians’ training only to save the patient’s life; and physicians’ narrow interpretation of when a patient is actually dying.
- Regarding physicians’ practice, strong evidence was found for physicians’ lack of confidence in taking responsibility for the care of the dying patient.
- These barriers need to be addressed to improve the quality of end-of-life care for patients and their families. Next to the physicians’ perspectives, it is important to examine the barriers related and reported by patients and their relatives, as well as other health care providers, in the ICU.

## Additional file

Additional file 1:
**PubMed search strategy for MEDLINE.**

## Abbreviations

GP: General physician
ICU: Intensive care unit
QI: Quality indicator
RWJF: Robert Wood Johnson Foundation
