# Supplementary material for: Physician-related barriers to communication and patient- and family-centred decision-making towards the end of life in intensive care: a systematic review
Source: Crit Care. 2014 Nov 18;18(6):604. doi: 10.1186/s13054-014-0604-z (PMC4258302; doi:10.1186/s13054-014-0604-z)
Supplement: Additional file 1: — PubMed search strategy for MEDLINE. [file 13054_2014_604_MOESM1_ESM.docx]

**Additional file 1**

**Pubmed search strategy for Medline***

**Physician-related barriers to communication and patient and family-centred decision making towards the end of life in intensive care: a systematic review.**

**Mieke Visser, Luc Deliens, Dirk Houttekier.**

(((("physicians"[MeSH] OR physician*[TIAB] OR doctor*[TIAB] OR intensivist*[TIAB])) AND ("Palliative Care"[Mesh] OR "Terminal Care"[Mesh] OR "Terminally ill"[Mesh] OR "Advance Care Planning"[Mesh] OR "palliative care"[TIAB] OR "Palliative treatment"[TIAB] OR "Palliative treatment"[TIAB] OR "Palliative treatments"[TIAB] OR "Palliative therapy"[TIAB] OR "Palliative medicine"[TIAB] OR "Palliative surgery"[TIAB] OR "Terminal care"[TIAB] OR "End of life care"[TIAB] OR "Euthanasia"[TIAB] OR "Mercy killing"[TIAB] OR "Mercy killings"[TIAB] OR "Hospice care"[TIAB] OR "Hospice Program"[TIAB] OR "Hospice Programs"[TIAB] OR "Bereavement Care"[TIAB] OR "Resuscitation orders"[TIAB] OR "Resuscitation order"[TIAB] OR "Withholding Resuscitation"[TIAB] OR "Resuscitation Policies"[TIAB] OR "Resuscitation Policy"[TIAB] OR "Do-Not-Resuscitate Orders"[TIAB] OR "Do-Not-Resuscitate Order"[TIAB] OR "Resuscitation Decisions"[TIAB] OR "Resuscitation Decision"[TIAB] OR "Assisted Suicides"[TIAB] OR "Assisted Death"[TIAB] OR "Assisted Deaths"[TIAB] OR "Assisted Suicide"[TIAB] OR "Supportive care"[TIAB] OR "Terminally ill"[TIAB] OR "Advance care planning"[TIAB] OR "Advance health care planning"[TIAB] OR "Advance Medical Planning"[TIAB] OR "Advance Directives"[TIAB] OR "Advance Directive"[TIAB] OR "Power of Attorney"[TIAB] OR "Living Wills"[TIAB] OR "Living Will"[TIAB])) AND ("Critical Care"[Mesh] OR "Intensive Care Units"[Mesh] OR "Critical care"[TIAB] OR "Intensive care"[TIAB])) AND (English[Language] OR Dutch[Language]) AND ( ( "2003/01/01"[PDat] : "2013/08/01"[PDat] ) )

*The search strategies for the other electronic databases (EMBASE, CINAHL, PsycINFO) we searched were derived from the Pubmed search Strategy for Medline.
